# Mr. Rule, on the Prevention of Bilious Fever

**Published:** 1801-10

**Authors:** J. Rule

**Affiliations:** Surgeon. Penryn, Cornwall


### *To the Editors of the Medical and Phyſical Journal.*

GENTLEMEN,

WHATEVER may be the opinion of gentlemen reſpecting the irritative property of Antimonium Tartarizatum I ſhall not attempt to diſpute, neither can I deny the reality of a fact ſo generally known and acknowledged; yet, when we come to a knowledge knowledge of any hidden property in medicine, it behoves us to make public the difcovery, that thereby we may be enabled to account for the repeated failure of operation.

We frequently find in adminiftering this medicine in dofes intended to prove emetic, our expectations prove abortive, for inftead of the expected effect it paffes off either by the bowels or infenfible perfpiration, leaving the ftomach in a ftate of undifturbed quiet.

Knowing this from repeated obfervation, I thought it might prove of ufe in allaying naufea, for which purpofe, in the year 1798, I tried its effect on fome foldiers then at Montego Bay in Jamaica, with unlooked-for fuccefs. And I flatter myfelf in having thereby prevented in feveral inftances the approach of fever, fo often preceded by irritation at the ftomach, which, neglected, terminates in exceffive puking and inflammation. I generally gave it on the earlieft approach of uneafy fenfation, but never after irritation became in any manner alarming.

What confirmed me in its fuccefs was the vifible diminution of numbers on the fick-lift, until I was myfelf attacked with yellow fever. Afterwards they became more fickly; but as I had only attended in the abfence of their furgeon, and he had returned, I had no further opportunity of then repeating the medicine preventively, and I have never given it as a cure.

My mode of prefcription was to mix carefully in a glafs mortar, three grains of antimonium tartarizatum in two ounces of good Spanifh white wine, of which the patient took a table fpoonful every fifth hour until naufea went off, which generally happened after the fecond or third fpoonful. He was afterwards put to bed, and made dilute with warm mint tea or thin gruel to bring on a moderate diaphorefis. This generally had the defired effect, and the patient returned to his duty on the following day, free from all complaint.

I have fince tried it, and when called in time, am proud to fay have but feldom failed in faving my patient; for when the antimonial folution had not the defired effect of allaying naufea, I never continued it fo as to increafe irritation, but inftantly had recourfe to the tepid bath, and other modes of cure.

I hope this may prove of fervice to my fellow-creatures in refcuing them, as it were, from the brink of the grave; and am, &c.

*Penryn, Cornwall,*

*Sept.* 5, 1801.

J. RULE, Surgeon.

